# Phylogeographic Analyses Strongly Suggest Cryptic Speciation in the Giant Spiny Frog (Dicroglossidae: *Paa spinosa*) and Interspecies Hybridization in *Paa*

**DOI:** 10.1371/journal.pone.0070403

**Published:** 2013-07-31

**Authors:** Shupei Ye, Hua Huang, Rongquan Zheng, Jiayong Zhang, Guang Yang, Shixia Xu

**Affiliations:** 1 Institute of Ecology, Zhejiang Normal University, Jinhua, Zhejiang, China; 2 Jiangsu Key Laboratory for Biodiversity and Biotechnology, College of Life Sciences, Nanjing Normal University, Nanjing, China; University of Western Ontario, Canada

## Abstract

Species identification is one of the most basic yet crucial issues in biology with potentially far-reaching implications for fields such as conservation, population ecology, and epidemiology. The widely distributed but threatened frog *Paa spinosa* has been speculated to represent a complex of multiple species. In this study, 254 individuals representing species of the genus *Paa* were investigated along the entire range of *P. spinosa*: 196 specimens of *P. spinosa*, 8 specimens of *P. jiulongensis*, 5 specimens of *P. boulengeri*, 20 specimens of *P. exilispinosa*, and 25 specimens of *P. shini*. Approximately 1333 bp of mtDNA sequence data (genes 12S rRNA and 16S rRNA) were used. Phylogenetic analyses were conducted using maximum parsimony, maximum likelihood and Bayesian inference. BEAST was used to estimate divergence dates of major clades. Results suggest that *P. spinosa* can be divided into three distinct major lineages. Each major lineage totally corresponds to geographical regions, revealing the presence of three candidate cryptic species. Isolation and differentiation among lineages are further supported by the great genetic distances between the lineages. The bifurcating phylogenetic pattern also suggests an east–west dispersal trend during historic cryptic speciation. Dating analysis estimates that *P. spinosa* from Western China split from the remaining *P. spinosa* populations in the Miocene and that *P. spinosa* from Eastern China diverged from Central China in the Pliocene. We also found that *P. exilispinosa* from Mainland China and Hong Kong might have a complex of multiple species. After identifying cryptic lineages, we then determine the discrepancy between the mtDNA and the morphotypes in several individuals. This discrepancy may have been caused by introgressive hybridization between *P. spinosa* and *P. shini*.

## Introduction

Biologists usually categorize species by morphology as the original tool [1], and morphological traits remain immensely important for taxonomic description and identification of species in the field. However speciation is not always accompanied by a morphological change. In the last decade, cryptic species have been revealed in most types of organism from fungi, insects, and mammals to other types by using molecular data [2], [3]. The actual number of biological species is likely to exceed the current tally of nominal species, most of which are delineated on purely morphological grounds [4]. Identification and characterization of the world’s numerous cryptic species complexes have become an ongoing challenge for evolutionary biologists with potentially far-reaching implications for fields such as biodiversity conservation [4], epidemiology [5], [6], and biological control [7], [8].

Molecular phylogenies and DNA sequence data are important for identification and discovery of cryptic biological diversity that usually cannot be distinguished by traditional non-genetic approaches [4], [9]. Genetic markers provide critical information regarding evolutionary relationships even in instances of morphological homogeneity, and even few markers can clearly demarcate species boundaries when groups are strongly diverged. A number of studies have revealed cryptic diversity among species of anurans by molecular genetic approaches in the last decade [10], [11], [12].

Chinese montane forests are among the world’s major centers that harbor large numbers of species relative to their extremely mountainous terrain [13]. Frogs have species recognition and mate choice systems that rely on non-morphological characteristics, which can be storehouses of vast cryptic diversity [12]. With these factors considered, a taxonomic understanding of anurans that live in Chinese mountain area is essential for future conservation and management decisions. The giant spiny frog *Paa spinosa* is characterized by keratinized skin spines on the chest in males. The males also develop hypertrophied forearms during the breeding season [14]. These characteristics are viewed as adaptations to males grasping females during amplexus [14], [15]. *P. spinosa* is a widespread native species of amphibians in the southern and southeast part of China. This species inhabits rocky streams in evergreen forests and open countryside on hills and mountains from 500 m to 1500 m above sea level [16], [17]. Since ancient times the thinking of ‘food as medicine’ has existed in Chinese medical theories and Chinese food therapy [18], and this species are used to be considered have both dietary and medicinal functions [19]. The wild populations of this species have been significantly decreasing because of ecological and environmental degradation as well as consumption as food.

Specific habitat requirements suggest that *P. spinosa* is likely to be a species complex and high genetic divergences among populations because of isolated population and poor overland dispersers. Che et al. [20]suggested that *P. spinosa* is likely a species complex rather than a single species according to nuclear and mitochondrial DNA sequence data, but fewer specimens (only seven specimens from four localities) were used to infer the phylogenetic relationships. Elucidating various aspects of this diversity, such as cryptic species and degree of endemism, is critical in the understanding and formation of geographic models of speciation for these montane regions. However, population genetics and the evolutionary history of *P*. *spinosa* have not been systematically investigated. In the present study, a large sample of specimens collected across the whole species range of *P. spinosa* was investigated using two mtDNA genes (12S rRNA, 16S rRNA). This study aimed to (1) test whether cryptic species existed within *P. spinosa*, (2) examine population genetic structure and phylogeographic patterns, (3) present a hypothesis of population dynamics explaining the current distribution and phylogeographic pattern, and (4) provide genetic information to further develop effective conservation recommendations for this economically important but threatened animal.

## Materials and Methods

### Ethics Statement

All frogs were released to the wild at their site of capture after tissue sampling. Tissue samples were preserved in 95% ethanol before they were deposited at Zhejiang Normal University under voucher numbers identified by locality-haplotype numbers. Our experimental procedures complied with the current laws on animal welfare and research in China, and were specifically approved by the Animal Research Ethics Committee of Zhejiang Normal University (Permit No. AREC 20061024). The Provincial Forestry Departments of Anhui, Fujian, Guangdong, Guangxi, Hunan, Jiangxi, Yunnan and Zhejiang provided permits for capturing frogs in China.

### Taxon Sampling and DNA Sequence Data

**Figure 1:**
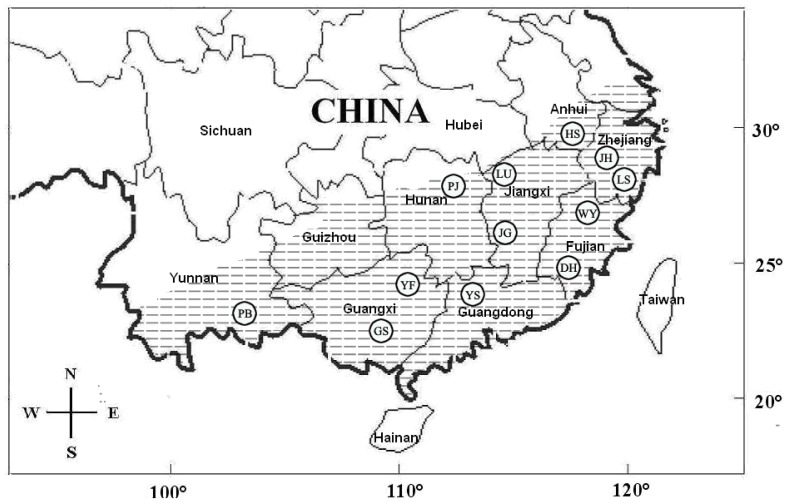
Map of sampling localities for this study. The hatched area represents the distribution of *Paa spinosa*. Twelve localities are grouped by city, with the following codes: DH, Dehua; GS, Longsheng; HS, Huangshan; JG, Jinggangshan; JH, Jinhua; PB, Pingbian; PJ, Pingjiang; LS, Lishui; LU, Lushan; WY, Wuyishan; YF, Yongfu; YS, Yangshan.

A total of 196 *P. spinosa* individuals were collected from 12 localities across South China (Table 1, Fig. 1). In addition, 8 individuals of *P. jiulongensis*, 5 individuals of *P. boulengeri*, 20 individuals of *P. exilispinosa*, and 25 individuals of *P. shini* were sampled and used as the ingroup. Moreover, the 12S rRNA and 16S rRNA genes previously reported as orthologous sequences for the *Paa* species were added to the dataset for subsequent phylogenetic analysis (Details are included in Table S1). Based on the work by Frost et al. [21], this study used the species *Fejervarya limnocharis* and *F*. *cancrivora* as the outgroups. Sequences of these two species were then obtained from GenBank.

**Table 1:** GPS coordinates and sample sizes for each geographic location.

Location	Coordinates	*P. spinosa*	*P. exilispinosa*	*P. boulengeri*	*P. jiulongensis*	*P. shini*
Jinhua	E119°37′12″, N29°6′36″	21	0	0	0	0
Lishui	E119°32′24″, N28°16′12″	21	0	0	0	0
Wuyishan	E118°0′36″, N27°16′12″	23	13	0	8	0
Dehua	E118°11′23″, N25°40′19″	21	7	0	0	0
Lushan	E116°13′19″, N29°40′06″	13	0	0	0	12
Jinggangshan	E114°6′12″, N26°20′24″	19	0	2	0	0
Pingjiang	E113°34′48″, N28°43′12″	10	0	1	0	0
Yangshan	E113°34′48″, N28°43′12″	15	0	2	0	0
Yongfu	E109°58′48″, N24°58′48″	20	0	0	0	0
Longsheng	E109°58′48″, N25°49′12″	4	0	0	0	13
Pingbian	E103°36′51″, N 22°48′36″	6	0	0	0	0
Total		196	20	5	8	25

Genomic DNA was extracted from muscle and toe clips by standard 3-step phenol/chloroform extraction [22]. Polymerase chain reaction (PCR) was conducted to amplify fragments of the mitochondrial genes 12S rRNA and 16S rRNA in a 50 µl volume reaction under the following conditions: initial denaturation for 4 min at 95°C, 35 cycles of denaturation for 1 min at 95°C, annealing for 1 min at 58°C, extension for 1 min at 72°C; final extension for 10 min at 72°C. 16S rRNA gene was amplified with primers 16Sar 5′-CGCCTGTTTACCAAAAACAT-3′ and 16Sbr 5′-CCGGTYTGAACTCAGATCAYGT-3′ [23]; 12S rRNA gene was amplified with primers 12SF 5′- AGTGCTGAAAACGCTAAGAC -3′ and 12SR 5′- AGGGCGACGGGCGGTGTGTAC -3′ [24], [25]. PCR products were purified using the Wizard PCR Preps DNA Purification Kit (Promega). Each fragment was sequenced in both directions by using the PCR primers. To confirm the identity of the amplified targets, sequence were submitted to BLAST searches before deposition in GenBank.

### Data Analysis

Sequences were aligned for each gene by using Clustal X 1.83 [26], and alignment was verified by eye with Bioedit 7.0.0 [27]. We calculated the number of polymorphic sites and haplotypes, as well as haplotype and nucleotide diversity for each taxon and each population by using DnaSP 4.0 [28].

Phylogenetic relationships among haplotypes were estimated using maximum parsimony (MP), maximum likelihood (ML), and Bayesian methods. Heuristic MP search with random-addition sequences and tree bisection and reconnection branch swapping in PAUP*4.0 [29] were conducted. The robustness of these analyses was assessed by non-parametric bootstrap replications using PAUP*4.0 with 100 replicates. ML analyses were performed by heuristic search methods conducted using PAUP*4.0 [29] with the general time-reversible (GTR+I+G) model of DNA evolution having the shape parameter of a gamma distribution (G). The GTR+I+G model was identified as the best-fitting model under the Akaike information criterion (AIC) as implemented in Modeltest 3.7 [30]; In the Bayesian analysis, we used MrBayes 3.1.2 [31] under the GTR+I+G model. BI used four simultaneous Metropolis-coupled Markov Chain Monte Carlo (MCMC) for 6,000,000 generations, we sampled a tree every 100 generations and calculated a consensus topology for 30,000 trees after discarding the first 30,000 trees (burn-in = 3,000,000). We regarded bootstrap values of 70% or greater for MP and ML, and Bayesian posterior probability of 0.95 as indicative of strong support [32], [33].

Species delimitation was guided by phylogenetic relationships based on molecular evidence [34], [35], [36]. The net between and ingroup mean distances between all major mtDNA clades were calculated following the Tamura and Nei (1993) distance module in MEGA 4.0 [37].

We used the BEAST version 1.6.1 [38] to construct an ultrametric tree and erect 95% confidence intervals for node heights. A molecular clock and previously published dates for *Paa* were used to estimate divergence times. We assumed a substitution rate ranging from 0.2% to 0.5% per Myr for the 12S and 16S genes, which are common in frogs [39], [40], [41], [42]. To estimate divergence times based on previously suggested dates, we calibrated our phylogeny by using one published divergence time to the most recent common ancestor (TMRCA) between the *P. boulengeri* and *P. shini* [43]. Fossil records and biogeographic inference were used to obtain the TMRCA of *P. boulengeri* and *P. shini* at about 27 Myr. Two MCMC runs were performed, with each running 20 million generations and logging parameters every 2000 generations. A burn-in of 10% was selected after likelihood score parameters were inspected in TRACER for stability.

## Results

### Sequence Characteristics

The 254 specimens were sequenced for 12S rRNA and 16S rRNA fragments,and new sequences were deposited in NCBI (Accession nos. JX989290-989797). Total alignment consisted of 1333 bp (12S = 805 bp; 16S = 528 bp) for the dataset in which 430 sites were variable and 372 sites were parsimony-informative. Sequences of the 12S rRNA fragment from previous studies [20], [21], [44] were shorter than those obtained in out study; thus, one region with 51 bp of 12S rRNA was excluded for further analysis. A total of 87 haplotypes were identified among the 254 ingroup sequences (Table S1).

### Genetic Distances

Uncorrected distances for 12S rRNA to 16S rRNA between all clades ranged from 1.7% to 9.2% (Table 2). All haplotypes from *P. spinosa* were considered collectively. The mean genetic distance between *P. spinosa* and other species is 4.7%, and that between clades C (*P. spinosa*) and D (*P. exilispinosa*) is 1.7%. Notably, the genetic distances between the clades of *P. spinosa* ranged from 3% to 8.7%.

**Table 2:** Numbers below the diagonal are genetic diversity within and between clades from mtDNA data, assessed by K2P distances (median, %).

Clade	Out	A	B	C	D	E	F	G
A	11.4(1.0)	0.3(0.3)						
B	12.7(1.1)	4.9(0.9)	0.6(0.5)					
C	11.9(1.1)	6.8(0.8)	8.5(0.9)	1.3(0.2)				
D	11.6(1.0)	6.7(0.8)	8.6(0.9)	1.7(0.3)	1.0(0.2)			
E	11.6(1.0)	6.8(0.8)	8.7(0.9)	3.0(0.4)	2.9(0.4)	1.8(0.8)		
F	12.2(1.1)	5.3(0.7)	7.9(0.9)	4.8(0.4)	4.8(0.7)	4.9(0.6)	0.8(0.8)	
G	11.6(1.0)	7.3(0.8)	9.2(0.9)	6.9(0.7)	7.0(0.7)	6.4(0.7)	6.6(0.7)	2.6(0.3)

Standard error estimates are shown within brackets.

### Phylogenetic Relationships

**Figure 2:**
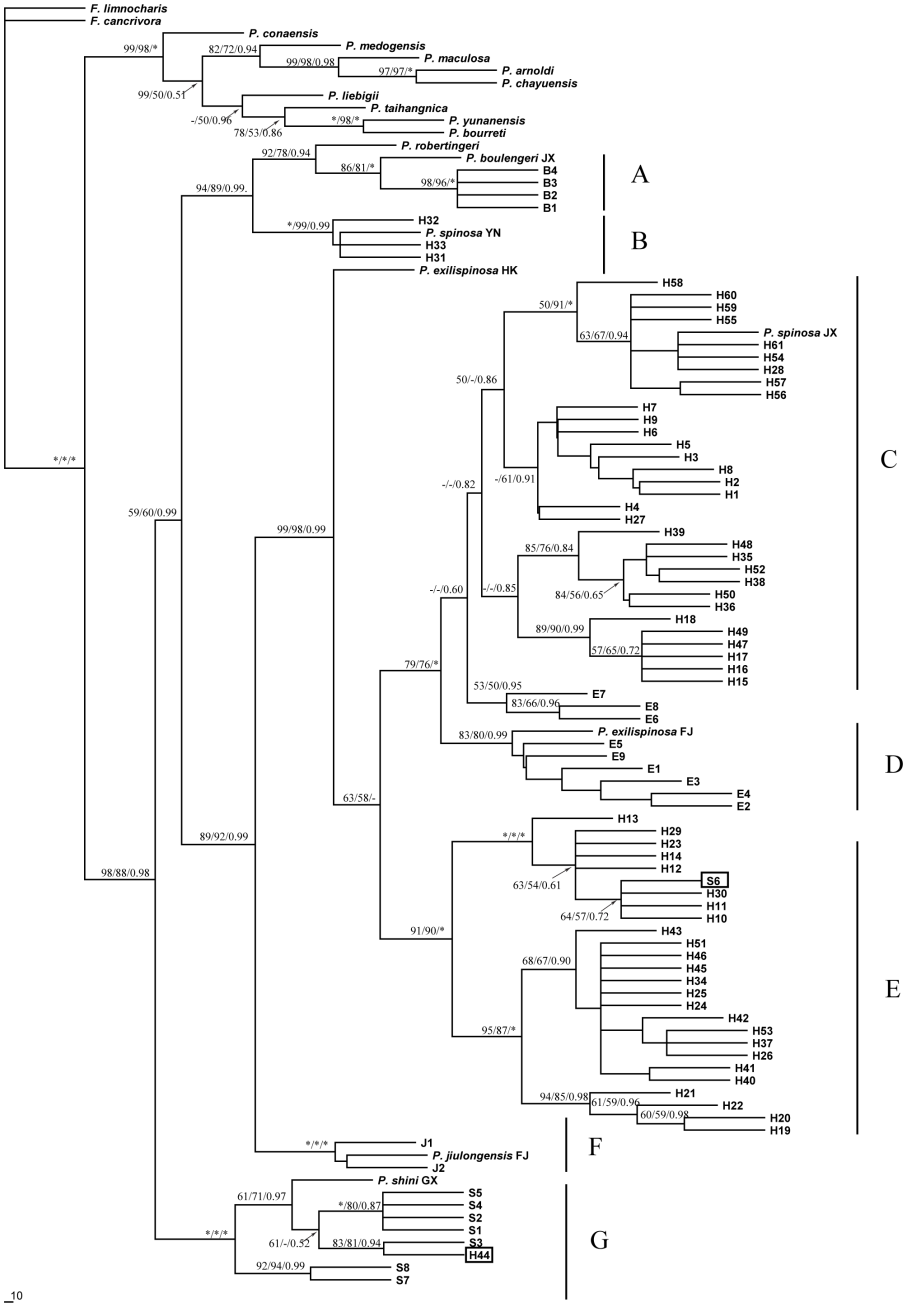
Phylogenetic relationships of haplotypes, geographic distribution, and frequency of each *Paa* haplotype. Phylogenetic relationships of haplotypes based on the 12S and 16S gene sequences, as determined by MP, ML, and Bayesian inference. Numbers above and below the branches are Bootstrap support values and Bayesian posterior probabilities ≥50 are indicated by the nodes. Legend: asterisk (“*”) indicates 100% ML and MP bootstrap support and 1.0 Bayesian posterior probabilities. “B,” haplotypes of *P. boulengeri*; “H,” haplotypes of *P. spinosa;* “J,” haplotypes of *P. jiulongensis*; “E,” haplotypes of *P. exilispinosa*; “S,” haplotypes of *P. shini*. *P. spinosa* YN and *P. spinosa* JX, sequences of *P. spinosa* obtained from GenBank; *P. exilispinosa* HK and *P. exilispinosa* FJ, sequence of *P. exilispinosa* obtained from GenBank; *P. jiulongensis* FJ, sequence of *P. jiulongensis* obtained from GenBank; *P. shini* GX, sequence of *P. shini* obtained from GenBank; *P. taihangnica*, *P. liebigii*, *P. conaensis*, *P. medogensis*, *P. maculosa*, *P. arnoldi*, *P. chayuensis*, *P. robertingeri*, *P. yunanensis*, *P. bourreti*, sequence of *P. taihangnica*, *P. liebigii*, *P. conaensis*, *P. medogensis*, *P. maculosa*, *P. arnoldi*, *P. chayuensis*, *P. robertingeri*, *P. yunanensis*, *P. bourreti* obtained from GenBank respectively.

MP, BI, and ML analyses generated almost identical tree topology and we present only the topology of ML in Figure 2. Phylogeny exhibits two quite well supported major lineages, and the clade composed of the subgenus *Quasipaa* (Clades A–G) is the sister taxon to the other species in *Paa*. Most relationships within these deep clades were resolved (Fig. 2).

Seven major haplotype clades were revealed in our study with strongly support (Fig. 2). Clade A includes all haplotypes of *P. boulengeri* from Guangdong, Hunan, Jiangxi, and Hubei provinces. Clade D includes *P. exilispinosa* from Fujian and Guangxi provinces, except three haplotype of *P. exilispinosa* E6, E7, E8. The haplotype of *P. exilispinosa* from Hong Kong was diverged far from Clade D. Clade F includes all haplotypes of *P. jiulongensis*. Clade G includes all haplotypes of *P. shini*, except *P. spinosa* H44, which possessed an mtDNA haplotype of *P. shini*. Within the *P. spinosa* complex, all haplotypes were clustered into three major groups, each of which corresponded to a geographical region. Clade B was found to be *P. spinosa* limited to Yunnan Province (Western China) known as the sister taxon of *P. boulengeri*, which formed a basal branch with a strong support. Clade C includes *P. spinosa* from Jiangxi, Hunan, Guangdong, and Guangxi provinces as well as Dehua in Fujian Province (Center and Southern China). Clade E includes *P. spinosa* from Anhui, Zhejiang, and Jiangxi provinces as well as Mt. Wuyi in Fujian Province (Southeast China). Interestingly, the haplotype *P. shini* S6 was also found in Clade E (Fig. 2).

### Diagnostic Sites

**Figure 3:**
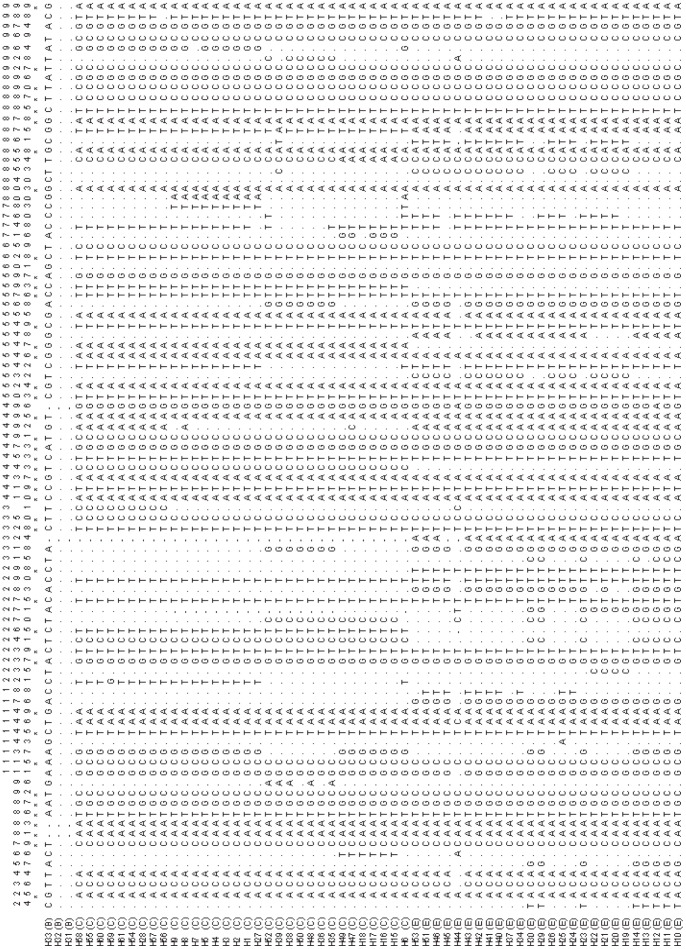
Variable sites of 12S and 16S mtDNA fragment in the three clades of *P. spinosa*, show the diagnostic sites. Numbers above sequences correspond to base positions of bariable sites. Haplotype names and B, C, E within brackets correspond to those clade in Figure 2. A dot(.) indicates identity with the top sequence. Hyphens(−)indicate gaps. Asterisks(*) indicate diagnostic sites.

Thirty diagnostic sites which could clearly distinguish the three major clades of *P. spinosa* were found (Fig. 3). For example, the special base composition at sites 25, 92,115, 237, and so on could differentiate the alleles H31–33 from the clade B (western China clade). Diagnostic sites were also found in the haplotypes from clade C at site 265, 443, 548, 593, and the “T” at site 298 only occurred in the haplotypes from clade E (central China clade) (Fig. 3).

### Estimation of Divergence Times

**Figure 4:**
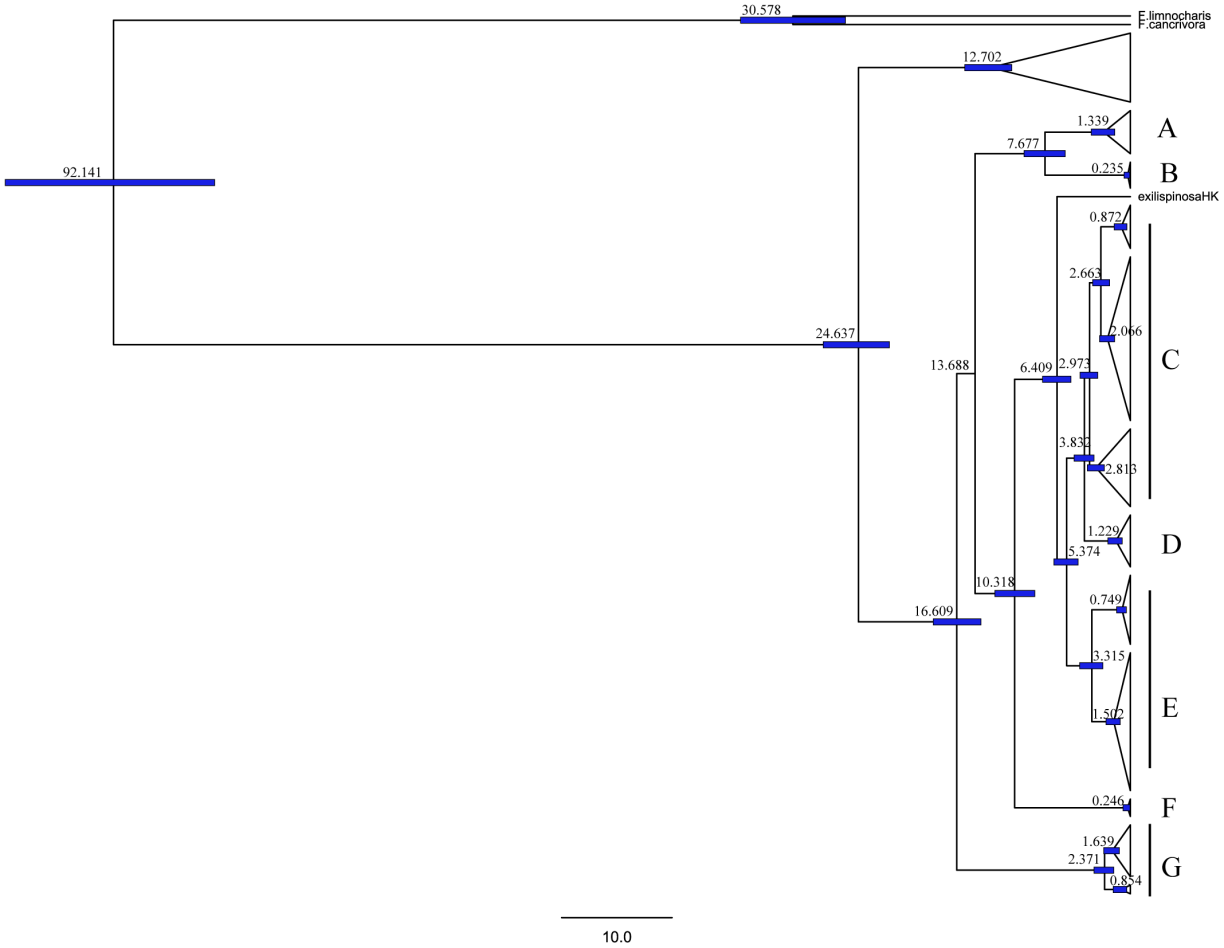
Time tree for all species in our study. Divergence time is shown on the node bars. Tree topology is derived from BEAST. Clades A, B, C, D, E, F, G correspond to those in Figure 2.

Estimated divergence times among the three clades of *P. spinosa* are shown in Fig. 4. Our estimates indicated that Clade B (*P. spinosa* from western China) separated from the remaining *P. spinosa* populations at approximately 14 Ma. Clades C (*P. spinosa* from Central China), D (*P. exilispinosa* from Mainland China), and E (*P. spinosa* from Eastern China) diverged between 5 and 3 Ma. *P. exilispinosa* from Hong Kong diverged at 6 Ma.

## Discussion

### Cryptic Species Complex

Taxonomic decisions are usually based on recognizable morphological characteristics. However, different species cannot always be recognized by phenotypic changes [4]. In this study, we apply the mtDNA tree-based method as a phylogenetic criterion to propose species boundaries considering geographically separated mtDNA clades as different phylogenetic species. Our data revealed three divergent lineages of *P*. *spinosa*, which allows us to propose the existence of three candidate cryptic species within different localities of *P*. *spinosa*. Furthermore, each lineage corresponds to specific sampling regions (Figs. 1 and 2). Clade B mainly corresponds to southern China; Clade C mainly corresponds to Central China (Guangdong, Guangxi, Hunan, and Jiangxi provinces and one in Fujian Province); Clade E exclusively corresponds to Southeast China. A previous study showed widely distributed *P*. *spinosa*, and Fujian is designated as the place of origin [45]. This study suggests that a higher species diversity is observed in *P*. *spinosa* than in one widely distributed species in previous studies.

The isolation and differentiation among the clades are further supported by genetic distances. In this study, the genetic distances among the three lineages were found to range from 3.9% to 8.7%. The distance between Clade C (consisting of *P*. *spinosa*) and Clade D (consisting of *P. exilispinosa*) was markedly larger (Table 2). Vences and Wake [46] indicated that interspecific genetic distances ranged from 1% to 16.5% for 16S rRNA sequences among the species of Mantellidae from Madagascar. The interspecific distances between the recognized genus *Engystomops* also ranged from 2.9% to 4.1% for the 12S rRNA and 16S rRNA mtDNAs [47]. Notably, genetic distances in *P*. *spinosa* were greater than those in other phylogenetic and taxonomic studies of amphibians. Although species delimitation using mtDNA only is controversial, data collected from our research strongly suggest that cryptic species are hidden within *P*. *spinosa*. This result also agrees with the suggestion that seven specimens of *P*. *spinosa* from four localities do not belong to one lineage, based on nuclear and mtDNA sequence in the study by Che et al. [20]. Though relationship between some *P. exilispinosa* (E6, E7, E8) and Clade C is still have confusion in this study (Fig. 2) and the relationship between *P*. *spinosa* and *P. exilispinosa* are not resolved in the study of Che et al. [20]. The evidence from genetic data and phylogenetic analysis suggest that Clade E should be designated as the type material for *P*. *spinosa* in the results of our study.

Amphibians are considered philopatric, showing a high degree of population subdivision over relatively short geographical distances [48], [49], [50], [51], [52]. The most common mode of amphibian species formation is supposed to be allopatric speciation [46]. Che et al. [43] demonstrated that the genus *Paa* originated from Indochina, and rapid divergences accompanied by geographical and probably paleoclimatic change occurred. In the present study, a west–east dispersal trend of *P*. *spinosa* populations was revealed. The Western China lineage contains samples only distributed in Yunan Province. The Central China lineage contains samples distributed from Guangxi and Hunan provinces to Jiangxi and Fujian provinces. The Eastern China lineage ranges from Jiangxi Province to Zhejiang Province. This high correspondence is not difficult to interpret because these frogs live in isolated montane streams.

### Evidence of Hybridization and Cryptic Lineages among *Paa*

Hybridization is prevalent in rapidly radiating groups, and amphibians and bird species remain adequately compatible to produce viable laboratory hybrids [53]. The clearest sign of introgression is the sympatric sharing of geographically localized mtDNA sequence haplotypes between otherwise genetically and morphologically divergent species [54]. In our study, 1 of 23 *P. shini* individuals (LUS1, Table S1) is identified as a hybrid that has the mtDNA haplotype of *P*. *spinosa*. Moreover, 1 *P*. *spinosa* (WY18) has an mtDNA haplotype of *P. shini* (Fig. 2). Despite the absence of hybridizable pairs of species known in Paini, intraspecific hybridization has occurred among different lineages of *P. boulengeri* because of secondary contact [55]. Considering that *Paa* is not an old genus composed through the Oligocene into the Miocene [43], the phylogenetic position of the haplotype S6 within Clade E and the haplotype H44 within Clade G most likely results from reciprocal introgression of mtDNA that occurred between these two amphibian species. In the present study, we also uncovered a population where haplotype Central China lineage and Eastern China lineage of *P*. *spinosa* coexisted in the Mt. Lushan area. Hence, Mt. Lushan is likely a rather narrow contact zone between populations of *P*. *spinosa*.

The haplotypes of *P. exilispinosa* HK from Hong Kong, which are sequences obtained from GenBank, are divided far from Mainland China. The separation of *P. exilispinosa* HK from mainland China started in the Pliocene. *P. exilispinosa* may also be a species complex rather than a single species; the formation of Hong Kong may explain this species complex. However no samples from Hong Kong was presented. Further systematic sampling from more geographic areas and more molecular markers should be used in future investigations to confirm the present hypothesis.

### Implications for Conservation

A recent worldwide effort to evaluate the conservation status of amphibians indicated that more than 400 species (including *P*. *spinosa*) are rapidly declining and may be considered threatened in the near future [56], [57]. Failure to diagnose biological diversity can hamper conservation efforts and basic scientific inquiry [58]. Thus, precise and correct species delimitation is essential given that the species are the basic units of analysis in biogeography, ecology, macroevolution, biodiversity assessment, as well as conservation and management. Widespread high-density species are less likely to succumb to extinction compared with small isolated species [59]. Our study suggests that the threatened *P. spinosa* should at least be divided into three species, with each characterized by the following: a smaller population, more restricted geographic ranges, greater vulnerability to further population decline, and extinction.

Hybrid zones represent a potentially important source of information regarding taxon divergence and population interaction [60]. Protections of the remaining populations have to be prioritized considering the limited resources. Protection of contact zones such as Lushan and Dehua, which have greater gene diversity, should be prioritized considering that various anthropogenic pressures such as commercial overexploitation and habitat destruction have threatened this species complex in the past decades.

## Supporting Information

Table S1 **Sampling information in** ***Paa*** **including specimens ID, localities, haplotypes and GenBank accession numbers.**
(DOC)
