# Supplementary material for: Phylogeographic Analyses Strongly Suggest Cryptic Speciation in the Giant Spiny Frog (Dicroglossidae: Paa spinosa) and Interspecies Hybridization in Paa
Source: PLoS One. 2013 Jul 31;8(7):e70403. doi: 10.1371/journal.pone.0070403 (PMC3729840; doi:10.1371/journal.pone.0070403)
Supplement: Table S1 — Sampling information in Paa including specimens ID, localities, haplotypes and GenBank accession numbers. (DOC) [file pone.0070403.s001.doc]

**Table S1** Sampling information in *Paa* including specimens ID, localities, haplotypes and GenBank accession numbers.

| Specimen ID | | Locality | Coordinates | Haplotype | GenBank Accession Nos. mtDNA | | |
| --- | --- | --- | --- | --- | --- | --- | --- |
|  |  |  |  |  | 12S | 16S | |
| Outgroups | | | | | | | |
| Genus *Fejervarya* | | | | | | | |
| *F. limnocharis* | | China: Sanya, Hainan | No data | *F. limnocharis* | EU979731  EU979788 | EU979848 | |
| *F. cancrivora* | | No data | No data | *F. cancrivora* | NC012647 | NC012647 | |
| Ingroups | | | | | | | |
| Genus *Paa* | | | | | | | |
| *P. arnoldi* | China: Pian ma, Lushui  Co., Yunnan | | No data | *P. arnoldi* | EU979714  EU979778 | EU979838 | |
| *P. chayuensis* | China: Shama Village,  Zayü Co., Xizang | | No data | *P. chayuensis* | EU979708  DQ118466 | DQ118510 | |
| *P. conaensis* | China: Mama, Cona  Co., Xizang | | No data | *P. conaensis* | EU979703  EU979774 | EU979834 | |
| *P. liebigii* | China: Yakong Co., Xizang | | No data | *P. liebigii* | EU979702  DQ118455 | DQ118499 | |
| *P. maculosa* | China: Xinmin,  Jingdong Co., Yunnan | | No data | *P. maculosa* | EU979707  DQ118468 | DQ118512 | |
| *P. medogensis* | China: 62 k, Medôg  Co., Xizang | | No data | *P. medogensis* | EU979704  DQ118463 | DQ118507 | |
| *P. robertingeri* | China: Zihuai, Hejiang  Co., Sichuan | | No data | *P. robertingeri* | EU979671  EU979754 | EU979814 | |
| *P. taihangnica* | China: Taihangshan,  Jiyuan, Henan | | No data | *P. taihangnica* | EU979724  EU979782 | EU979842 | |
| *P. yunnanensis* | China: Dongping, Qiaojia Co., Yunnan | | No data | *P. yunnanensis* | EU979684  EU979763 | EU979823 | |
| *P. bourreti* | Vietnam: Sa Pa vicinity, Lao Cai | | No data | *P. bourreti* | EU979689  EU979767 | EU979827 | |
| *P. boulengeri* |  | |  |  |  |  | |
| *P. boulengeri* HB | China: Yichang, Hubei | | No data | *P. boulengeri* HB | EU979672  EU979755 | EU979815 | |
| JGB1 | China:Jinggangshan,Jiangxi | | E114°6′12″, N26°20′24″ | B1 | JX989597 | JX989343 | |
| JGB2 | China:Jinggangshan, Jiangxi | | E114°6′12″, N26°20′24″ | B1 | JX989598 | JX989344 | |
| PJB1 | China: Pingjiang Hunan | | E113°34′48″,N28°43′12″ | B2 | JX989599 | JX989345 | |
| YSB1 | China:Yangshan Guangdong | | E112°37′48″,N24°28′48″ | B3 | JX989600 | JX989346 | |
| YSB2 | China:Yangshan Guangdong | | E112°37′48″,N24°28′48″ | B4 | JX989601 | JX989347 | |
| *p. jiulongensis* | | | | | | | |
| *P. jiulongensis* FJ | | China:Sangang, Fujian | No data | *P. jiulongensis*FJ | EU979651 DQ118441 | | DQ118485 |
| WYJ1 | | China:Wuyishan, Fujian | E118°0′36″, N27°16′12″ | J1 | JX989569 | | JX989315 |
| WYJ2 | | China:Wuyishan, Fujian | E118°0′36″, N27°16′12″ | J2 | JX989570 | | JX989316 |
| WYJ3 | | China:Wuyishan, Fujian | E118°0′36″, N27°16′12″ | J2 | JX989571 | | JX989317 |
| WYJ4 | | China:Wuyishan, Fujian | E118°0′36″, N27°16′12″ | J2 | JX989572 | | JX989318 |
| WYJ5 | | China:Wuyishan, Fujian | E118°0′36″, N27°16′12″ | J2 | JX989573 | | JX989319 |
| WYJ6 | | China:Wuyishan, Fujian | E118°0′36″, N27°16′12″ | J2 | JX989574 | | JX989320 |
| WYJ7 | | China:Wuyishan, Fujian | E118°0′36″, N27°16′12″ | J2 | JX989575 | | JX989321 |
| WYJ8 | | China:Wuyishan, Fujian | E118°0′36″, N27°16′12″ | J2 | JX989576 | | JX989322 |
| *P. shini* | | | | | | | |
| *P. shini* GX | China:Longsheng,Guangxi | | No data | *P. shini* GX | EU979653 DQ118442 | | DQ118486 |
| GSS1 | China:Longsheng, Guangxi | | E109°58′48″,N25°49′12″ | S1 | JX989544 | | JX989290 |
| GSS2 | China:Longsheng, Guangxi | | E109°58′48″,N25°49′12″ | S2 | JX989545 | | JX989291 |
| GSS3 | China:Longsheng, Guangxi | | E109°58′48″,N25°49′12″ | S3 | JX989546 | | JX989292 |
| GSS4 | China:Longsheng, Guangxi | | E109°58′48″,N25°49′12″ | S3 | JX989547 | | JX989293 |
| GSS5 | China:Longsheng, Guangxi | | E109°58′48″,N25°49′12″ | S3 | JX989548 | | JX989294 |
| GSS6 | China:Longsheng, Guangxi | | E109°58′48″,N25°49′12″ | S4 | JX989549 | | JX989295 |
| GSS7 | China:Longsheng, Guangxi | | E109°58′48″,N25°49′12″ | S5 | JX989550 | | JX989296 |
| GSS8 | China:Longsheng, Guangxi | | E109°58′48″,N25°49′12″ | S3 | JX989551 | | JX989297 |
| GSS9 | China:Longsheng, Guangxi | | E109°58′48″,N25°49′12″ | S3 | JX989552 | | JX989298 |
| GSS10 | China:Longsheng, Guangxi | | E109°58′48″,N25°49′12″ | S3 | JX989553 | | JX989299 |
| GSS11 | China:Longsheng, Guangxi | | E109°58′48″,N25°49′12″ | S3 | JX989554 | | JX989300 |
| GSS12 | China:Longsheng, Guangxi | | E109°58′48″,N25°49′12″ | S3 | JX989555 | | JX989301 |
| GSS13 | China:Longsheng, Guangxi | | E109°58′48″,N25°49′12″ | S3 | JX989556 | | JX989302 |
| LUS1 | China: Lushan, Jiangxi | | E116°13′19″,N29°40′06″ | S6 | JX989557 | | JX989303 |
| LUS2 | China: Lushan, Jiangxi | | E116°13′19″,N29°40′06″ | S7 | JX989558 | | JX989304 |
| LUS3 | China: Lushan, Jiangxi | | E116°13′19″,N29°40′06″ | S7 | JX989559 | | JX989305 |
| LUS4 | China: Lushan, Jiangxi | | E116°13′19″,N29°40′06″ | S7 | JX989560 | | JX989306 |
| LUS5 | China: Lushan, Jiangxi | | E116°13′19″,N29°40′06″ | S8 | JX989561 | | JX989307 |
| LUS6 | China: Lushan, Jiangxi | | E116°13′19″,N29°40′06″ | S7 | JX989562 | | JX989308 |
| LUS7 | China: Lushan, Jiangxi | | E116°13′19″,N29°40′06″ | S7 | JX989563 | | JX989309 |
| LUS8 | China: Lushan, Jiangxi | | E116°13′19″,N29°40′06″ | S7 | JX989564 | | JX989310 |
| LUS9 | China: Lushan, Jiangxi | | E116°13′19″,N29°40′06″ | S7 | JX989565 | | JX989311 |
| LUS10 | China: Lushan, Jiangxi | | E116°13′19″,N29°40′06″ | S7 | JX989566 | | JX989312 |
| LUS11 | China: Lushan, Jiangxi | | E116°13′19″,N29°40′06″ | S7 | JX989567 | | JX989313 |
| LUS12 | China: Lushan, Jiangxi | | E116°13′19″,N29°40′06″ | S7 | JX989568 | | JX989314 |
| *P. exilispinosa* | | | | | | | |
| *P. exilispinosa* FJ | China: Sangang Fujian | | No data | *P. exilispinosa* FJ | EU979648 DQ118440 | | DQ118484 |
| *P. exilispinosa* HK | China: Hong Kong | | E114°11′, N22°15′ | *P. exilispinosa* HK | EU979649 EU979739 | | EU979799 |
| WYE1 | China: Wuyishan, Fujian | | E118°0′36″, N27°16′12″ | E1 | JX989584 | | JX989330 |
| WYE2 | China: Wuyishan, Fujian | | E118°0′36″, N27°16′12″ | E4 | JX989585 | | JX989331 |
| WYE3 | China: Wuyishan, Fujian | | E118°0′36″, N27°16′12″ | E2 | JX989586 | | JX989332 |
| WYE4 | China: Wuyishan, Fujian | | E118°0′36″, N27°16′12″ | E2 | JX989587 | | JX989333 |
| WYE5 | China: Wuyishan, Fujian | | E118°0′36″, N27°16′12″ | E3 | JX989588 | | JX989334 |
| WYE6 | China: Wuyishan, Fujian | | E118°0′36″, N27°16′12″ | E3 | JX989589 | | JX989335 |
| WYE7 | China: Wuyishan, Fujian | | E118°0′36″, N27°16′12″ | E3 | JX989590 | | JX989336 |
| WYE8 | China: Wuyishan, Fujian | | E118°0′36″, N27°16′12″ | E3 | JX989591 | | JX989337 |
| WYE9 | China: Wuyishan, Fujian | | E118°0′36″, N27°16′12″ | E4 | JX989592 | | JX989338 |
| WYE10 | China: Wuyishan, Fujian | | E118°0′36″, N27°16′12″ | E5 | JX989593 | | JX989339 |
| WYE11 | China: Wuyishan, Fujian | | E118°0′36″, N27°16′12″ | E5 | JX989594 | | JX989340 |
| WYE12 | China: Wuyishan, Fujian | | E118°0′36″, N27°16′12″ | E5 | JX989595 | | JX989341 |
| WYE13 | China: Wuyishan, Fujian | | E118°0′36″, N27°16′12″ | E5 | JX989596 | | JX989342 |
| DHE1 | China: DeHua, Fujian | | E118°11′23″,N25°40′19″ | E6 | JX989577 | | JX989323 |
| DHE2 | China: DeHua, Fujian | | E118°11′23″,N25°40′19″ | E7 | JX989578 | | JX989324 |
| DHE3 | China: DeHua, Fujian | | E118°11′23″,N25°40′19″ | E6 | JX989579 | | JX989325 |
| DHE4 | China: DeHua, Fujian | | E118°11′23″,N25°40′19″ | E9 | JX989580 | | JX989326 |
| DHE5 | China: DeHua, Fujian | | E118°11′23″,N25°40′19″ | E6 | JX989581 | | JX989327 |
| DHE6 | China: DeHua, Fujian | | E118°11′23″,N25°40′19″ | E8 | JX989582 | | JX989328 |
| DHE7 | China: DeHua, Fujian | | E118°11′23″,N25°40′19″ | E8 | JX989583 | | JX989329 |
| *P. spinosa* | | | | | | | |
| *P. spinosa* YN | China: Pingbian, Yunnan | | No data | *P. spinosa* YN | EU979667 DQ118436 | | DQ118480 |
| *P. spinosa* JX | China: Pingbian, Yunnan | | No data | *P. spinosa* JX | EU979667 DQ118436 | | DQ118480 |
| DH1 | China: DeHua, Fujian | | E118°11′23″,N25°40′19″ | H1 | JX989764 | | JX989523 |
| DH2 | China: DeHua, Fujian | | E118°11′23″,N25°40′19″ | H2 | JX989765 | | JX989524 |
| DH3 | China: DeHua, Fujian | | E118°11′23″,N25°40′19″ | H3 | JX989766 | | JX989525 |
| DH4 | China: DeHua, Fujian | | E118°11′23″,N25°40′19″ | H4 | JX989767 | | JX989526 |
| DH5 | China: DeHua, Fujian | | E118°11′23″,N25°40′19″ | H5 | JX989768 | | JX989527 |
| DH6 | China: DeHua, Fujian | | E118°11′23″,N25°40′19″ | H2 | JX989769 | | JX989528 |
| DH7 | China: DeHua, Fujian | | E118°11′23″,N25°40′19″ | H9 | JX989770 | | JX989529 |
| DH8 | China: DeHua, Fujian | | E118°11′23″,N25°40′19″ | H6 | JX989771 | | JX989530 |
| DH9 | China: DeHua, Fujian | | E118°11′23″,N25°40′19″ | H4 | JX989772 | | JX989531 |
| DH10 | China: DeHua, Fujian | | E118°11′23″,N25°40′19″ | H2 | JX989773 | | JX989532 |
| DH11 | China: DeHua, Fujian | | E118°11′23″,N25°40′19″ | H2 | JX989774 | | JX989533 |
| DH12 | China: DeHua, Fujian | | E118°11′23″,N25°40′19″ | H2 | JX989775 | | JX989534 |
| DH13 | China: DeHua, Fujian | | E118°11′23″,N25°40′19″ | H5 | JX989776 | | JX989535 |
| DH14 | China: DeHua, Fujian | | E118°11′23″,N25°40′19″ | H2 | JX989777 | | JX989536 |
| DH15 | China: DeHua, Fujian | | E118°11′23″,N25°40′19″ | H2 | JX989778 | | JX989537 |
| DH16 | China: DeHua, Fujian | | E118°11′23″,N25°40′19″ | H2 | JX989779 | | JX989538 |
| DH17 | China: DeHua, Fujian | | E118°11′23″,N25°40′19″ | H8 | JX989780 | | JX989539 |
| DH18 | China: DeHua, Fujian | | E118°11′23″,N25°40′19″ | H3 | JX989781 | | JX989540 |
| DH19 | China: DeHua, Fujian | | E118°11′23″,N25°40′19″ | H1 | JX989782 | | JX989541 |
| DH20 | China: DeHua, Fujian | | E118°11′23″,N25°40′19″ | H7 | JX989783 | | JX989542 |
| DH21 | China: DeHua, Fujian | | E118°11′23″,N25°40′19″ | H2 | JX989784 | | JX989543 |
| HS1 | China: Huangshan, Anhui | | E118°34′12″,N30°4′12″ | H10 | JX989680 | | JX989426 |
| HS2 | China: Huangshan, Anhui | | E118°34′12″,N30°4′12″ | H10 | JX989681 | | JX989427 |
| HS3 | China: Huangshan, Anhui | | E118°34′12″,N30°4′12″ | H10 | JX989682 | | JX989428 |
| HS4 | China: Huangshan, Anhui | | E118°34′12″,N30°4′12″ | H12 | JX989683 | | JX989429 |
| HS5 | China: Huangshan, Anhui | | E118°34′12″,N30°4′12″ | H11 | JX989684 | | JX989430 |
| HS6 | China: Huangshan, Anhui | | E118°34′12″,N30°4′12″ | H13 | JX989685 | | JX989431 |
| HS7 | China: Huangshan, Anhui | | E118°34′12″,N30°4′12″ | H13 | JX989686 | | JX989432 |
| HS8 | China: Huangshan, Anhui | | E118°34′12″,N30°4′12″ | H11 | JX989687 | | JX989433 |
| HS9 | China: Huangshan, Anhui | | E118°34′12″,N30°4′12″ | H10 | JX989688 | | JX989434 |
| HS10 | China: Huangshan, Anhui | | E118°34′12″,N30°4′12″ | H11 | JX989689 | | JX989435 |
| HS11 | China: Huangshan, Anhui | | E118°34′12″,N30°4′12″ | H11 | JX989690 | | JX989436 |
| HS12 | China: Huangshan, Anhui | | E118°34′12″,N30°4′12″ | H13 | JX989691 | | JX989437 |
| HS13 | China: Huangshan, Anhui | | E118°34′12″,N30°4′12″ | H10 | JX989692 | | JX989438 |
| HS14 | China: Huangshan, Anhui | | E118°34′12″,N30°4′12″ | H14 | JX989693 | | JX989439 |
| HS15 | China: Huangshan, Anhui | | E118°34′12″,N30°4′12″ | H10 | JX989694 | | JX989440 |
| HS16 | China: Huangshan, Anhui | | E118°34′12″,N30°4′12″ | H13 | JX989695 | | JX989441 |
| HS17 | China: Huangshan, Anhui | | E118°34′12″,N30°4′12″ | H13 | JX989696 | | JX989442 |
| HS18 | China: Huangshan, Anhui | | E118°34′12″,N30°4′12″ | H14 | JX989697 | | JX989443 |
| HS19 | China: Huangshan, Anhui | | E118°34′12″,N30°4′12″ | H11 | JX989698 | | JX989444 |
| HS20 | China: Huangshan, Anhui | | E118°34′12″,N30°4′12″ | H12 | JX989699 | | JX989445 |
| HS21 | China: Huangshan, Anhui | | E118°34′12″,N30°4′12″ | H13 | JX989700 | | JX989446 |
| HS22 | China: Huangshan, Anhui | | E118°34′12″,N30°4′12″ | H13 | JX989701 | | JX989447 |
| HS23 | China: Huangshan, Anhui | | E118°34′12″,N30°4′12″ | H14 | JX989702 | | JX989448 |
| JG1 | China: Jinggangshan, Jiangxi | | E114°6′12″, N26°20′24″ | H15 | JX989739 | | JX989491 |
| JG2 | China: Jinggangshan, Jiangxi | | E114°6′12″, N26°20′24″ | H15 | JX989740 | | JX989492 |
| JG3 | China: Jinggangshan, Jiangxi | | E114°6′12″, N26°20′24″ | H16 | JX989741 | | JX989493 |
| JG4 | China: Jinggangshan, Jiangxi | | E114°6′12″, N26°20′24″ | H15 | JX989742 | | JX989494 |
| JG5 | China: Jinggangshan, Jiangxi | | E114°6′12″, N26°20′24″ | H15 | JX989743 | | JX989495 |
| JG6 | China: Jinggangshan, Jiangxi | | E114°6′12″, N26°20′24″ | H18 | JX989744 | | JX989496 |
| JG7 | China: Jinggangshan, Jiangxi | | E114°6′12″, N26°20′24″ | H15 | JX989745 | | JX989497 |
| JG8 | China: Jinggangshan, Jiangxi | | E114°6′12″, N26°20′24″ | H15 | JX989746 | | JX989498 |
| JG9 | China: Jinggangshan, Jiangxi | | E114°6′12″, N26°20′24″ | H18 | JX989747 | | JX989499 |
| JG10 | China: Jinggangshan, Jiangxi | | E114°6′12″, N26°20′24″ | H17 | JX989748 | | JX989500 |
| JG11 | China: Jinggangshan, Jiangxi | | E114°6′12″, N26°20′24″ | H16 | JX989749 | | JX989501 |
| JG12 | China: Jinggangshan, Jiangxi | | E114°6′12″, N26°20′24″ | H15 | JX989750 | | JX989502 |
| JG13 | China: Jinggangshan, Jiangxi | | E114°6′12″, N26°20′24″ | H16 | JX989751 | | JX989503 |
| JG14 | China: Jinggangshan, Jiangxi | | E114°6′12″, N26°20′24″ | H18 | JX989752 | | JX989504 |
| JG15 | China: Jinggangshan, Jiangxi | | E114°6′12″, N26°20′24″ | H18 | JX989753 | | JX989505 |
| JG16 | China: Jinggangshan, Jiangxi | | E114°6′12″, N26°20′24″ | H17 | JX989754 | | JX989506 |
| JG17 | China: Jinggangshan, Jiangxi | | E114°6′12″, N26°20′24″ | H18 | JX989755 | | JX989507 |
| JG18 | China: Jinggangshan, Jiangxi | | E114°6′12″, N26°20′24″ | H15 | JX989756 | | JX989508 |
| JG19 | China: Jinggangshan, Jiangxi | | E114°6′12″, N26°20′24″ | H16 | JX989757 | | JX989509 |
| JH1 | China: Jinhua, Zhejiang | | E119°37′12″,N29°6′36″ | H19 | JX989703 | | JX989449 |
| JH2 | China: Jinhua, Zhejiang | | E119°37′12″,N29°6′36″ | H20 | JX989704 | | JX989450 |
| JH3 | China: Jinhua, Zhejiang | | E119°37′12″,N29°6′36″ | H22 | JX989705 | | JX989451 |
| JH4 | China: Jinhua, Zhejiang | | E119°37′12″,N29°6′36″ | H22 | JX989706 | | JX989452 |
| JH5 | China: Jinhua, Zhejiang | | E119°37′12″,N29°6′36″ | H23 | JX989707 | | JX989453 |
| JH6 | China: Jinhua, Zhejiang | | E119°37′12″,N29°6′36″ | H22 | JX989708 | | JX989454 |
| JH7 | China: Jinhua, Zhejiang | | E119°37′12″,N29°6′36″ | H22 | JX989709 | | JX989455 |
| JH8 | China: Jinhua, Zhejiang | | E119°37′12″,N29°6′36″ | H20 | JX989710 | | JX989456 |
| JH9 | China: Jinhua, Zhejiang | | E119°37′12″,N29°6′36″ | H22 | JX989711 | | JX989457 |
| JH10 | China: Jinhua, Zhejiang | | E119°37′12″,N29°6′36″ | H19 | JX989712 | | JX989458 |
| JH11 | China: Jinhua, Zhejiang | | E119°37′12″,N29°6′36″ | H22 | JX989713 | | JX989459 |
| JH12 | China: Jinhua, Zhejiang | | E119°37′12″,N29°6′36″ | H20 | JX989714 | | JX989460 |
| JH13 | China: Jinhua, Zhejiang | | E119°37′12″,N29°6′36″ | H22 | JX989715 | | JX989461 |
| JH14 | China: Jinhua, Zhejiang | | E119°37′12″,N29°6′36″ | H22 | JX989716 | | JX989462 |
| JH15 | China: Jinhua, Zhejiang | | E119°37′12″,N29°6′36″ | H21 | JX989717 | | JX989463 |
| JH16 | China: Jinhua, Zhejiang | | E119°37′12″,N29°6′36″ | H20 | JX989718 | | JX989464 |
| JH17 | China: Jinhua, Zhejiang | | E119°37′12″,N29°6′36″ | H22 | JX989719 | | JX989465 |
| JH18 | China: Jinhua, Zhejiang | | E119°37′12″,N29°6′36″ | H22 | JX989720 | | JX989466 |
| JH19 | China: Jinhua, Zhejiang | | E119°37′12″,N29°6′36″ | H22 | JX989721 | | JX989467 |
| JH20 | China: Jinhua, Zhejiang | | E119°37′12″,N29°6′36″ | H22 | JX989722 | | JX989468 |
| JH21 | China: Jinhua, Zhejiang | | E119°37′12″,N29°6′36″ | H23 | JX989723 | | JX989469 |
| LS1 | China: Lishui, Zhejiang | | E119°32′24″,N28°16′12″ | H24 | JX989655 | | JX989401 |
| LS2 | China: Lishui, Zhejiang | | E119°32′24″,N28°16′12″ | H24 | JX989656 | | JX989402 |
| LS3 | China: Lishui, Zhejiang | | E119°32′24″,N28°16′12″ | H24 | JX989657 | | JX989403 |
| LS4 | China: Lishui, Zhejiang | | E119°32′24″,N28°16′12″ | H24 | JX989658 | | JX989404 |
| LS5 | China: Lishui, Zhejiang | | E119°32′24″,N28°16′12″ | H24 | JX989659 | | JX989405 |
| LS6 | China: Lishui, Zhejiang | | E119°32′24″,N28°16′12″ | H24 | JX989660 | | JX989406 |
| LS7 | China: Lishui, Zhejiang | | E119°32′24″,N28°16′12″ | H24 | JX989661 | | JX989407 |
| LS8 | China: Lishui, Zhejiang | | E119°32′24″,N28°16′12″ | H26 | JX989662 | | JX989408 |
| LS9 | China: Lishui, Zhejiang | | E119°32′24″,N28°16′12″ | H24 | JX989663 | | JX989409 |
| LS10 | China: Lishui, Zhejiang | | E119°32′24″,N28°16′12″ | H25 | JX989664 | | JX989410 |
| LS11 | China: Lishui, Zhejiang | | E119°32′24″,N28°16′12″ | H26 | JX989665 | | JX989411 |
| LS12 | China: Lishui, Zhejiang | | E119°32′24″,N28°16′12″ | H24 | JX989666 | | JX989412 |
| LS13 | China: Lishui, Zhejiang | | E119°32′24″,N28°16′12″ | H24 | JX989667 | | JX989413 |
| LS14 | China: Lishui, Zhejiang | | E119°32′24″,N28°16′12″ | H24 | JX989668 | | JX989414 |
| LS15 | China: Lishui, Zhejiang | | E119°32′24″,N28°16′12″ | H24 | JX989669 | | JX989415 |
| LS16 | China: Lishui, Zhejiang | | E119°32′24″,N28°16′12″ | H24 | JX989670 | | JX989416 |
| LS17 | China: Lishui, Zhejiang | | E119°32′24″,N28°16′12″ | H25 | JX989671 | | JX989417 |
| LS18 | China: Lishui, Zhejiang | | E119°32′24″,N28°16′12″ | H26 | JX989672 | | JX989418 |
| LS19 | China: Lishui, Zhejiang | | E119°32′24″,N28°16′12″ | H25 | JX989673 | | JX989419 |
| LS20 | China: Lishui, Zhejiang | | E119°32′24″,N28°16′12″ | H24 | JX989674 | | JX989420 |
| LS21 | China: Lishui, Zhejiang | | E119°32′24″,N28°16′12″ | H24 | JX989675 | | JX989421 |
| GS1 | China:Longsheng, Guangxi | | E109°58′48″,N25°49′12″ | H27 | JX989676 | | JX989422 |
| GS2 | China:Longsheng, Guangxi | | E109°58′48″,N25°49′12″ | H27 | JX989677 | | JX989423 |
| GS3 | China:Longsheng, Guangxi | | E109°58′48″,N25°49′12″ | H27 | JX989678 | | JX989424 |
| GS4 | China:Longsheng, Guangxi | | E109°58′48″,N25°49′12″ | H27 | JX989679 | | JX989425 |
| LU1 | China: Lushan, Jiangxi | | E116°13′19″,N29°40′06″ | H28 | JX989785 | | JX989510 |
| LU2 | China: Lushan, Jiangxi | | E116°13′19″,N29°40′06″ | H28 | JX989786 | | JX989511 |
| LU3 | China: Lushan, Jiangxi | | E116°13′19″,N29°40′06″ | H28 | JX989787 | | JX989512 |
| LU4 | China: Lushan, Jiangxi | | E116°13′19″,N29°40′06″ | H28 | JX989788 | | JX989513 |
| LU5 | China: Lushan, Jiangxi | | E116°13′19″,N29°40′06″ | H28 | JX989789 | | JX989514 |
| LU6 | China: Lushan, Jiangxi | | E116°13′19″,N29°40′06″ | H29 | JX989790 | | JX989515 |
| LU7 | China: Lushan, Jiangxi | | E116°13′19″,N29°40′06″ | H30 | JX989791 | | JX989516 |
| LU8 | China: Lushan, Jiangxi | | E116°13′19″,N29°40′06″ | H29 | JX989792 | | JX989517 |
| LU9 | China: Lushan, Jiangxi | | E116°13′19″,N29°40′06″ | H30 | JX989793 | | JX989518 |
| LU10 | China: Lushan, Jiangxi | | E116°13′19″,N29°40′06″ | H30 | JX989794 | | JX989519 |
| LU11 | China: Lushan, Jiangxi | | E116°13′19″,N29°40′06″ | H29 | JX989795 | | JX989520 |
| LU12 | China: Lushan, Jiangxi | | E116°13′19″,N29°40′06″ | H30 | JX989796 | | JX989521 |
| LU13 | China: Lushan, Jiangxi | | E116°13′19″,N29°40′06″ | H29 | JX989797 | | JX989522 |
| PB1 | China: Pingbian, Ynnan | | E103°36′51″, N 22°48′36″ | H33 | JX989758 | | JX989485 |
| PB2 | China: Pingbian, Ynnan | | E103°36′51″, N 22°48′36″ | H31 | JX989759 | | JX989486 |
| PB3 | China: Pingbian, Ynnan | | E103°36′51″, N 22°48′36″ | H32 | JX989760 | | JX989487 |
| PB4 | China: Pingbian, Ynnan | | E103°36′51″, N 22°48′36″ | H31 | JX989761 | | JX989488 |
| PB5 | China: Pingbian, Ynnan | | E103°36′51″, N 22°48′36″ | H31 | JX989762 | | JX989489 |
| PB6 | China: Pingbian, Ynnan | | E103°36′51″, N 22°48′36″ | H31 | JX989763 | | JX989490 |
| PJ1 | China: Pingjiang, Hunan | | E113°34′48″, N28°43′12″ | H34 | JX989645 | | JX989391 |
| PJ2 | China: Pingjiang, Hunan | | E113°34′48″, N28°43′12″ | H34 | JX989646 | | JX989392 |
| PJ3 | China: Pingjiang, Hunan | | E113°34′48″, N28°43′12″ | H35 | JX989647 | | JX989393 |
| PJ4 | China: Pingjiang, Hunan | | E113°34′48″, N28°43′12″ | H36 | JX989648 | | JX989394 |
| PJ5 | China: Pingjiang, Hunan | | E113°34′48″, N28°43′12″ | H36 | JX989649 | | JX989395 |
| PJ6 | China: Pingjiang, Hunan | | E113°34′48″, N28°43′12″ | H35 | JX989650 | | JX989396 |
| PJ7 | China: Pingjiang, Hunan | | E113°34′48″, N28°43′12″ | H37 | JX989651 | | JX989397 |
| PJ8 | China: Pingjiang, Hunan | | E113°34′48″, N28°43′12″ | H39 | JX989652 | | JX989398 |
| PJ9 | China: Pingjiang, Hunan | | E113°34′48″, N28°43′12″ | H36 | JX989653 | | JX989399 |
| PJ10 | China: Pingjiang, Hunan | | E113°34′48″, N28°43′12″ | H38 | JX989654 | | JX989400 |
| WY1 | China: Wuyishan, Fujian | | E118°0′36″, N27°16′12″ | H40 | JX989602 | | JX989348 |
| WY2 | China: Wuyishan, Fujian | | E118°0′36″, N27°16′12″ | H41 | JX989603 | | JX989349 |
| WY3 | China: Wuyishan, Fujian | | E118°0′36″, N27°16′12″ | H40 | JX989604 | | JX989350 |
| WY4 | China: Wuyishan, Fujian | | E118°0′36″, N27°16′12″ | H40 | JX989605 | | JX989351 |
| WY5 | China: Wuyishan, Fujian | | E118°0′36″, N27°16′12″ | H40 | JX989606 | | JX989352 |
| WY6 | China: Wuyishan, Fujian | | E118°0′36″, N27°16′12″ | H41 | JX989607 | | JX989353 |
| WY7 | China: Wuyishan, Fujian | | E118°0′36″, N27°16′12″ | H40 | JX989608 | | JX989354 |
| WY8 | China: Wuyishan, Fujian | | E118°0′36″, N27°16′12″ | H42 | JX989609 | | JX989355 |
| WY9 | China: Wuyishan, Fujian | | E118°0′36″, N27°16′12″ | H43 | JX989610 | | JX989356 |
| WY10 | China: Wuyishan, Fujian | | E118°0′36″, N27°16′12″ | H42 | JX989611 | | JX989357 |
| WY11 | China: Wuyishan, Fujian | | E118°0′36″, N27°16′12″ | H40 | JX989612 | | JX989358 |
| WY12 | China: Wuyishan, Fujian | | E118°0′36″, N27°16′12″ | H41 | JX989613 | | JX989359 |
| WY13 | China: Wuyishan, Fujian | | E118°0′36″, N27°16′12″ | H41 | JX989614 | | JX989360 |
| WY14 | China: Wuyishan, Fujian | | E118°0′36″, N27°16′12″ | H41 | JX989615 | | JX989361 |
| WY15 | China: Wuyishan, Fujian | | E118°0′36″, N27°16′12″ | H43 | JX989616 | | JX989362 |
| WY16 | China: Wuyishan, Fujian | | E118°0′36″, N27°16′12″ | H42 | JX989617 | | JX989363 |
| WY17 | China: Wuyishan, Fujian | | E118°0′36″, N27°16′12″ | H41 | JX989618 | | JX989364 |
| WY18 | China: Wuyishan, Fujian | | E118°0′36″, N27°16′12″ | H44 | JX989619 | | JX989365 |
| WY19 | China: Wuyishan, Fujian | | E118°0′36″, N27°16′12″ | H45 | JX989620 | | JX989366 |
| WY20 | China: Wuyishan, Fujian | | E118°0′36″, N27°16′12″ | H45 | JX989621 | | JX989367 |
| WY21 | China: Wuyishan, Fujian | | E118°0′36″, N27°16′12″ | H46 | JX989622 | | JX989368 |
| WY22 | China: Wuyishan, Fujian | | E118°0′36″, N27°16′12″ | H45 | JX989623 | | JX989369 |
| WY23 | China: Wuyishan, Fujian | | E118°0′36″, N27°16′12″ | H47 | JX989624 | | JX989370 |
| YS1 | China:Yangshan, Guangdong | | E112°37′48″,N24°28′48″ | H48 | JX989724 | | JX989470 |
| YS2 | China:Yangshan, Guangdong | | E112°37′48″,N24°28′48″ | H49 | JX989725 | | JX989471 |
| YS3 | China:Yangshan, Guangdong | | E112°37′48″,N24°28′48″ | H51 | JX989726 | | JX989472 |
| YS4 | China:Yangshan, Guangdong | | E112°37′48″,N24°28′48″ | H48 | JX989727 | | JX989473 |
| YS5 | China:Yangshan, Guangdong | | E112°37′48″,N24°28′48″ | H50 | JX989728 | | JX989474 |
| YS6 | China:Yangshan, Guangdong | | E112°37′48″,N24°28′48″ | H50 | JX989729 | | JX989475 |
| YS7 | China:Yangshan, Guangdong | | E112°37′48″,N24°28′48″ | H50 | JX989730 | | JX989476 |
| YS8 | China:Yangshan, Guangdong | | E112°37′48″,N24°28′48″ | H51 | JX989731 | | JX989477 |
| YS9 | China:Yangshan, Guangdong | | E112°37′48″,N24°28′48″ | H53 | JX989732 | | JX989478 |
| YS10 | China:Yangshan, Guangdong | | E112°37′48″,N24°28′48″ | H50 | JX989733 | | JX989479 |
| YS11 | China:Yangshan, Guangdong | | E112°37′48″,N24°28′48″ | H48 | JX989734 | | JX989480 |
| YS12 | China:Yangshan, Guangdong | | E112°37′48″,N24°28′48″ | H52 | JX989735 | | JX989481 |
| YS13 | China:Yangshan, Guangdong | | E112°37′48″,N24°28′48″ | H50 | JX989736 | | JX989482 |
| YS14 | China:Yangshan, Guangdong | | E112°37′48″,N24°28′48″ | H50 | JX989737 | | JX989483 |
| YS15 | China:Yangshan, Guangdong | | E112°37′48″,N24°28′48″ | H52 | JX989738 | | JX989484 |
| YF1 | China: Yongfu, Guangxi | | E109°58′48″,N24°58′48″ | H55 | JX989625 | | JX989371 |
| YF2 | China: Yongfu, Guangxi | | E109°58′48″,N24°58′48″ | H61 | JX989626 | | JX989372 |
| YF3 | China: Yongfu, Guangxi | | E109°58′48″,N24°58′48″ | H54 | JX989627 | | JX989373 |
| YF4 | China: Yongfu, Guangxi | | E109°58′48″,N24°58′48″ | H59 | JX989628 | | JX989374 |
| YF5 | China: Yongfu, Guangxi | | E109°58′48″,N24°58′48″ | H60 | JX989629 | | JX989375 |
| YF6 | China: Yongfu, Guangxi | | E109°58′48″,N24°58′48″ | H56 | JX989630 | | JX989376 |
| YF7 | China: Yongfu, Guangxi | | E109°58′48″,N24°58′48″ | H55 | JX989631 | | JX989377 |
| YF8 | China: Yongfu, Guangxi | | E109°58′48″,N24°58′48″ | H55 | JX989632 | | JX989378 |
| YF9 | China: Yongfu, Guangxi | | E109°58′48″,N24°58′48″ | H54 | JX989633 | | JX989379 |
| YF10 | China: Yongfu, Guangxi | | E109°58′48″,N24°58′48″ | H60 | JX989634 | | JX989380 |
| YF11 | China: Yongfu, Guangxi | | E109°58′48″,N24°58′48″ | H56 | JX989635 | | JX989381 |
| YF12 | China: Yongfu, Guangxi | | E109°58′48″,N24°58′48″ | H60 | JX989636 | | JX989382 |
| YF13 | China: Yongfu, Guangxi | | E109°58′48″,N24°58′48″ | H61 | JX989637 | | JX989383 |
| YF14 | China: Yongfu, Guangxi | | E109°58′48″,N24°58′48″ | H60 | JX989638 | | JX989384 |
| YF15 | China: Yongfu, Guangxi | | E109°58′48″,N24°58′48″ | H57 | JX989639 | | JX989385 |
| YF16 | China: Yongfu, Guangxi | | E109°58′48″,N24°58′48″ | H60 | JX989640 | | JX989386 |
| YF17 | China: Yongfu, Guangxi | | E109°58′48″,N24°58′48″ | H58 | JX989641 | | JX989387 |
| YF18 | China: Yongfu, Guangxi | | E109°58′48″,N24°58′48″ | H54 | JX989642 | | JX989388 |
| YF19 | China: Yongfu, Guangxi | | E109°58′48″,N24°58′48″ | H55 | JX989643 | | JX989389 |
| YF20 | China: Yongfu, Guangxi | | E109°58′48″,N24°58′48″ | H61 | JX989644 | | JX989390 |
